# Cost-utility analysis of imrecoxib compared with diclofenac for patients with osteoarthritis

**DOI:** 10.1186/s12962-021-00275-7

**Published:** 2021-04-20

**Authors:** Xueshan Sun, Xuemei Zhen, Xiaoqian Hu, Yuanyuan Li, ShuYan Gu, Yuxuan Gu, Zixuan Zhao, Wei Yang, Hengjin Dong

**Affiliations:** 1 Center for Health Policy Studies, School of Public Health, Zhejiang University School of Medicine, Zijingang Campus, Rd 866 Yuhang, Xihu District, Hangzhou, Zhejiang China; 2 School of Health Care Management, Shandong University, 44 Wenhuaxi Rd., Jinan, Shandong China; 3 NHC Key Laboratory of Health Economics and Policy Research (Shandong University), 44 Wenhuaxi Rd., Jinan, Shandong China; 4 Center for Health Policy and Management Studies, School of Government, Nanjing University, Nanjing, Jiangsu China; 5 Shanghai Suvalue Health Scienfific Ltd., Shanghai, China; 6 The Fourth Affiliated Hospital Zhejiang University School of Medicine, Yiwu, Zhejiang China

**Keywords:** Cost-utility analysis, Imrecoxib, Diclofenac, Osteoarthritis, Proton pump inhibitor

## Abstract

**Background:**

To estimate the cost -utility of imrecoxib compared with diclofenac, as well as the addition of a proton pump inhibitor to both two treatment strategies, for patients with osteoarthritis, from a Chinese healthcare perspective.

**Methods:**

A Markov model was built. Costs of managing osteoarthritis and initial adverse events were collected from a Medical Database which collected information from 170 hospitals. Other parameters were obtained from the literature. Subgroup analyses were conducted for people at high risk of gastrointestinal or cardiovascular adverse events. Deterministic and probabilistic sensitivity analyses were performed.

**Results:**

Imrecoxib was highly cost-effective than diclofenac (the ICER was $401.58 and $492.77 in patients at low and high gastrointestinal and cardiovascular risk, respectively). The addition of a proton pump inhibitor was more cost -effective compared with single drug for both treatment strategies. Findings remained robust to sensitivity analyses. 59.04% and 57.16% probability for the co-prescription of imrecoxib and a proton pump inhibitor to be the most cost-effective strategy in all patients considered using the cost-effectiveness threshold of $30,000.

**Conclusions:**

The addition of a proton pump inhibitor to both imrecoxib and diclofenac was advised. Imrecoxib provides a valuable option for patients with osteoarthritis. Uncertainties existed in the model, and the suggestions can be adopted with caution.

**Supplementary Information:**

The online version contains supplementary material available at 10.1186/s12962-021-00275-7.

## Background

Osteoarthritis (OA) is a chronic disease with a high prevalence of 46.3% in Chinese who aged 40 years-old and above [1]. OA is associated with high disability rate, which can increase the incidence of cardiovascular disease (CV) and all-cause mortality rate, accounting for the main cause of disability in many countries [2]. According to the recommendations from both American Academy of Orthopedic Surgeons (AAOS) and Osteoarthritis Research Society International (OARSI), the use of Nonsteroidal anti-inflammatory drugs (NSAIDs) was highly recommended, which was also listed as first-line drugs managing OA in Chinese Guideline for Diagnosis and Treatment of OA [2–4]. There are two types of NSAIDs: traditional NSAIDs and newly developed selective COX-2 inhibitors. Similar efficacy of pain relief was found in both traditional NSAIDs and COX-2 inhibitors, while traditional NSAIDs were associated with gastrointestinal (GI) side effects and selective COX-2 inhibitors were developed to reduce GI adverse events [3, 5]. Meanwhile, cardiovascular (CV) adverse events were found in both traditional NSAIDs and COX-2 inhibitors.

Imrecoxib is a Chinese-patent COX-2 inhibitor, which was approved by the Chinese Food and Drug Administration (CFDA) in 2011. Diclofenac is a traditional NSAID, which was prescribed widely in managing OA. Imrecoxib was reported to have a lower rate of GI adverse events [6], but a higher drug price, the long-term cost-effectiveness of imrecoxib stay unknown. The National Institute for Health and Care Excellence (NICE) published a guideline (CG 59) for the management of OA in 2008 [7, 8]. In the guideline, the cost effectiveness of NSAIDs, selective COX-2 inhibitors, selective COX-2 inhibitors + proton pump inhibitor (PPI), and NSAIDs + PPI were compared. It drew up efficacy and safety data from three randomized controlled trials (RCTs): CLASS (celecoxib, ibuprofen, and diclofenac), MEDAL (etoricoxib and diclofenac), and TARGET (lumiracoxib, naproxen, and ibuprofen). It assumed that the NSAIDs included had same efficacy of pain relief when managing OA but with different GI and CV risks, and therefore had different cost-effectiveness. Furthermore, NICE also provided an OA model which was widely used in different regions to explore the cost-effective of drugs managing OA from the perspective of their local healthcare system, which provides an efficient way to conduct cost effectiveness for drugs managing OA [5, 9–11].

The objective of this study is to perform a cost utility analysis of imrecoxib and diclofenac, and also the addition of a PPI to both imrecoxib and diclofenac for patients with osteoarthritis. The model used in the present study was based on the OA model provided by the NICE, the analyses were conducted from the perspective of Chinese healthcare system, hoping to provide suggestions for relevant stakeholders.

## Methods

The model used in the present study is a cost-utility analysis based on the CG59 NICE OA model, which is a Markov model. The outcomes are increased cost, increased quality adjusted life years (QALYs), and incremental cost-effectiveness ratio (ICER). In addition, the present study was performed in accordance with the consolidated health economic evaluation reporting standards (CHEERS) (Additional file 1: Table S7). This study was approved by the institutional review board of Zhejiang University School of Public health, and no human subjects were involved.

### Comparators

In the present study, we compared the cost effectiveness of imrecoxib (100 mg twice a day, 100 mg BID) versus (vs.) diclofenac (50 mg three times a day, 50 mg TID), with and without the addition of omeprazole co-prescription (200 mg QD). Imrecoxib was chosen because it’s a relatively new selective COX-2 inhibitor developed in China in 2011, and its cost effectiveness was not fully known, which caused great interest on its cost-effectiveness in the healthcare system of China. Diclofenac is a widely-used traditional NSAID in managing OA. There’s necessity to compare the cost-effectiveness between the two drugs, to provide more suggestions for relevant departments in China when managing OA from the perspective of long-term cost-effectiveness. The addition of a PPI was considered to be more cost-effective than single drug in the NICE guideline, however, the cost-effectiveness of the addition to imrecoxib still stay unknown. Therefore, the addition of omeprazole to imrecoxib and diclofenac was also considered in the analysis, which was widely used as a PPI for patients with osteoarthritis.

### Model description

Both of the efficacy and safety of different treatment strategies were taken into consideration in the present model. The details of the model can be found in the NICE guideline (Additional file 1: Figure S1) [7, 8]. The health states that make up the Markov model represent a range of possible adverse events (AEs): GI symptoms, symptomatic ulcer, complicated GI, myocardial infarct (MI), stroke and heart failure (HF). Except for GI discomforts, other AEs are assumed to have continuing impact over the patients’ remaining lifetimes, therefore, there’re five post AE states: post symptomatic ulcer, post complicated GI, post MI, post stroke, and post HF. In addition, death and normal states without AEs are also included in the model. Each health state has associated cost and QALYs. It was assumed that once a patient has an AE (except for GI discomforts, because GI discomforts was supposed to be a minor AE and patients in that state don’t need to stop the medication), they would stop the medication and stay in that post state until dead.

The model is a lifetime model, and would be terminated if patients are 80-years-old or dead. Two groups with different ages (55 years-old and 65 years-old) were both estimated in line with different risks of AEs. The annual discount rate of both cost and utility was set to 5% according to the Chinese Guidelines of Pharmacoeconomics [12]. Half cycle corrections were made for both cost and QALY. The simulation was carried out initially for 100 cycles and 60 cycles with 3 months in each circle for patients at low and high GI and CV risk group, respectively. Cohort simulation with 100,000 patients per circle was performed in the base-case analyses, and 100,000 Monte Carlo simulations were performed in probability sensitivity analysis (PSA) analyses.

### Patients

The present model estimated results for OA patients aged 55 years-old and 65 years-old. Patients aged 55 years-old were assumed to have lower GI and CV risk, while patients aged 65 years-old were assumed to have higher GI and CV risk (a 2.96-times greater risk of developing an ulcer or complicated GI events, and 1.94-times greater risk of developing CV events) [8].

### Cost

Managing costs of OA and initial AEs were extracted from the Hospital Information System (HIS) of 170 hospitals through the Su-Value Database from 2016 to 2018 [13]. Data were extracted according to the ICD-10 code. It was assumed that the cost of managing OA was consisted of drug cost and other outpatient expense extracted from the Su-Value Database. Drug cost was calculated by using treatment duration in each circle, recommended dose and drug price, recommended dose and drug price were obtained from the Beijing Medicine Sunshine Purchasing System [14], and treatment duration in each circle was adjusted according to the consultation of doctors (Additional file 1: Table S1-3). In each circle, the cost of managing GI discomforts and symptomatic ulcer for all patients was supposed to include the cost of one outpatient visiting, while that of complicated GI, stroke, HF and MI were supposed to include one outpatient and inpatient visiting, which was similar to the assumption in the CG59 guideline (Additional file 1: Tables S4-5) [8]. It was supposed that there is no maintenance cost for GI events, while there’s a risk for patients who experienced GI events would suffer again, therefore the cost of post complicated GI and post symptomatic ulcer were calculated by multiplying the recurrence rate and the cost of initial AE states. When it comes to CV states, it was supposed that patients who suffered CV would have a maintenance cost to manage CV events. Because the cost of post CV states can’t be obtained from the Su-Value Database directly, therefore, maintenance cost of three post-CV states were obtained from literature which reported the cost in Chinese patients (Table 1). When patients cannot continue to take the medication of specific drug, the topical diclofenac was assumed to be adopted as a medication to manage OA [15] as the suggestion of the NICE OA model.

**Table 1:** Model parameters inputs

**Adverse events**							**Source**
	**GI discomforts**	**Symptomatic ulcer**	**Complicated GI**	**Stroke**	**MI**	**HF**	
*Cost ($)*^a^							
Initial states	10.52 + cost of medication of OA	34.57	1354.15	1289.92	5190.93	1182.33	SuValue Database (Additional fie 1: Table S4-5)
Post states	–	0.81^b^	21.54^b^	619.59	948.47	451.29	[13]
*Utility weights for AEs (1 = OA patients without any AEs)*							
Initial states	0.73	0.55	0.46	0.35	0.37	0.71	[7]
Post states	1.00	0.98	0.98	0.71	0.88	1.00	[7]
*Absolute AEs rates (%)*							
Diclofenac	21.30	0.14	0.07	0.06	0.09	0.02	[11]
*Relative risk of AEs rate of different treatment strategies*							
Celecoxib vs. diclofenac	0.66	0.43	0.68	0.51	1.40	1.42	[9]
Imrecoxib vs. celecoxib	0.50	1.36	0.50	1.00	1.00	1.00	[16, 17]
Imrecoxib vs. diclofenac	0.33	0.58	0.34	0.51	1.40	1.42	–
*Relative risk of AEs rate with the addition of a PPI*							
Diclofenac	0.43	0.37	0.46	1.00	1.00	1.00	[5]
imrecoxib	0.25	0.25	0.25	1.00	1.00	1.00	[5]

^a^RMB exchange rate against the USD was 100:689.85 in 2019, and the consumer price index (CPI) was 101.4%, 102.0%, 101.6%, 102.1% and 102.9% in 2015, 2016, 2017, 2018, and 2019, respectively [20]. All the cost was adjusted to 2019 based on the exchange rate and CPI

^b^ The maintenance cost of symptomatic ulcer and complicated GI were calculated by multiplying the cost of initial state by the recurrence rate, the recurrence rate were 2.33%[21] and 1.59%[22] of symptomatic ulcer and complicated GI in each circle, respectively

### Quality of life

QALYs was used to represent quality of life, due to the sparse data, the QALYs data were extracted from the NICE OA model. The QALYs of OA patients without any AEs were measured based on the efficacy of drug and the QALYs of OA symptom itself, it was assumed that all NSAIDs/selective COX-2 inhibitors were equally efficacious, which indicated that the QALYs of OA patients treated with NSAIDs/selective COX-2 inhibitors was higher than that of OA patients without any drug medication. The QALYs of initial and post AE states were also extracted (Table 1).

### Transition probabilities

There are RCTs comparing imrecoxib vs. celecoxib, celecoxib vs. diclofenac, while there’s no RCT comparing imrecoxib and diclofenac directly. Therefore, indirect comparison was conducted to obtain the imrecoxib relative risk of AEs compared to diclofenac. Absolute AEs rate of diclofenac was extracted from a meta-review which pooled the AEs of diclofenac observed in CLASS (celecoxib 800 mg, diclofenac 150 mg, ibuprofen 2400 mg), MEDAL (etoricoxib 73 mg, diclofenac 150 mg), EDGE (etoricoxib 90 mg, diclofenac 150 mg), and CONDOR (celecoxib 400 mg, diclofenac 150 mg) [11]. Celecoxib relative risk compared to diclofenac was obtained from a meta-review pooled the relative risk of AEs observed in CLASS and CONDOR, in which comparisons of celecoxib and diclofenac were conducted [9]. Imrecoxib relative risk compared to celecoxib was obtained from relevant literature, through literature review, two RCTs compared the safety of imrecoxib and celecoxib was included into analysis [16, 17]. The addition of a PPI can cause the reduction in the risk of GI-related AEs both in NSAIDs and selective COX-2 inhibitors, and the effect was obtained from literature, which reported the results of a meta-analysis [5]. The proportions of withdrawing due to GI symptoms were extracted from literature, which were 13.9% and 11.2% for NSAIDs and selective COX-2 inhibitors, respectively [8].

The observation period of the rate reported in the literature may not be consistent with the period divided in the model, thereby the probability was obtained by adjusting the instantaneous rate, the formula is [18]: r = −[In(1−P_1_)]/t_1_, P_2_ = 1−exp(−rt_2_), here r represents the instantaneous rate, P_1_ represents the rate observed in literature during specific period, P_2_ is the probability needed in the model, t_1_ is the time of observation in the literature while t_2_ is the time set in the model. The mortality rates of general population and patients with AEs were transmitted to probabilities using the formula (Additional file 1: Table S6).

### Sensitivity analysis

Deterministic sensitivity analysis (DSA) was performed by varying parameters to explore the robustness of the model and access the main influencing factors: discount rate varied from 0 to 8% according to Chinese Guidelines of Pharmacoeconomics [12]; parameters of cost, utility and possibility were set up ± 20%.

In addition to DSA, a PSA was also performed. It was required by the NICE updated guidance for technology assessment that all cost-effectiveness models submitted to the institute should use PSA [19]. DSA can only simultaneously analyze the impact of a limited number of input parameters on results (in the present study, the distributions of cost, probability and utility were set, Additional file 1: Table S8) [12]. When the model runs, a parameter for each input is randomly changed according to its preset distribution [7, 8], the mean cost and QALYs were obtained from the PSA results.

## Results

### Incremental cost-effectiveness ratio (ICER)

In the base-case analysis, no treatment strategy was strictly dominated by any other strategy. The addition of a PPI to both imrecoxib and diclofenac was cost-effective, the ICER of co-prescription of a PPI to imrecoxib was $8656.09 and $8178.07 per QALY in the low and high GI and CV risk group, respectively, the ICER of co-prescription of a PPI to diclofenac was $320.83 and $363.61 per QALY in the low and high GI and CV risk group, respectively. When it comes to single drug, imrecoxib was more cost-effective than diclofenac, with the ICER of $401.58 and $492.77 per QALY in the low and high GI and CV risk group, respectively. Meanwhile, the co-prescription of a PPI to imrecoxib was more cost-effective than the co-prescription of a PPI to diclofenac (The ICER was $8274.80 and $7011.67 in the low GI and CV risk groups, respectively) (Table 2).

**Table 2:** Incremental cost effectiveness ratios, mean results (all patients considered)

Treatment	Cost($)/per patient	QALYs/per patient	ICER (△C/△E)	Comparator
*Patients aged 55 years old (lower risk of GI and CV events)*				
Diclofenac	1298.73	4.16	–	–
Diclofenac + PPI	1767.47	5.63	320.83	Diclofenac
Imrecoxib	1894.66	5.65	401.58	Diclofenac
Imrecoxib + PPI	2181.21	5.68	8656.09	Imrecoxib
Imrecoxib + PPI	–	–	8274.80	Diclofenac + PPI
*Patients aged 65 years old (higher risk of GI and CV events)*				
Diclofenac	1373.86	3.37	–	–
Diclofenac + PPI	1773.89	4.47	363.61	Diclofenac
Imrecoxib	1927.92	4.50	492.77	Diclofenac
Imrecoxib + PPI	2194.59	4.53	8178.07	Imrecoxib
Imrecoxib + PPI	–	–	7011.67	Diclofenac + PPI

### Parameters influencing the ICERs

DSAs were performed for the base-case results for all patients considered. It showed that the main influencing factors of ICERs reported in the base-case results were risk of MI, discount rate of utility and cost, and utility of GI discomforts. Parameters related to MI were the important influencing factors, includes relative risk of probability of MI (imrecoxib vs. diclofenac, NSAIDs + PPI vs. NSAIDs, selective COX-2 inhibitors + PPI vs. selective COX-2 inhibitors), cost of post-MI (Additional fie 1: Figure S2–9).

Although uncertainties exist in the present model with the wide range of parameters, it was found that the base-case results were robust to the sensitivity analysis, most of the ICERs below $10,000 (1.0 GDP per capita approximately), while the ICER of imrecoxib + PPI vs. imrecoxib, imrecoxib + PPI vs. diclofeanc + PPI exceed $10,000 but below $15,000 in all the patients considered (Additional fie 1: Figure S2–9).

### Probabilistic representation of uncertainty

**Fig. 1:**
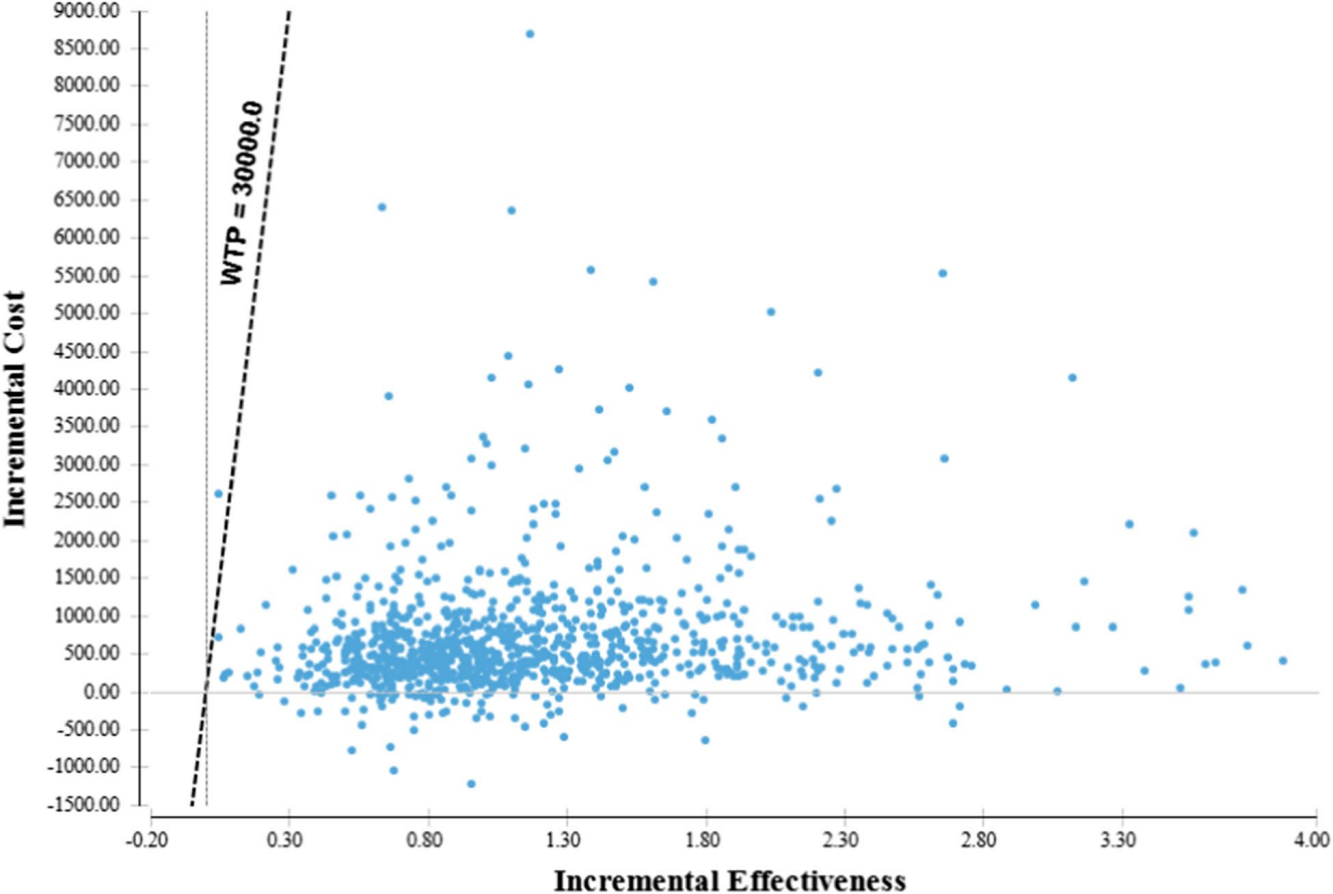
Cost effectiveness scatterplot (Imrecoxib vs. Diclofenac, low gastrointestinal and cardiovascular risk)

**Fig. 2:**
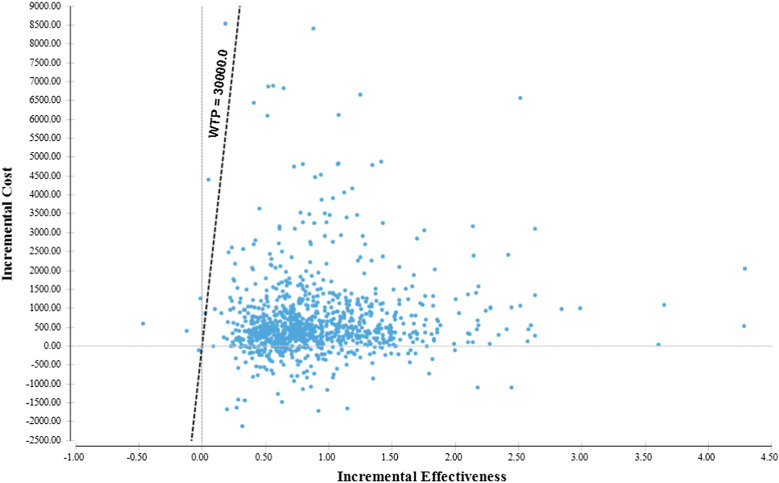
Cost effectiveness scatterplot (Imrecoxib vs. Diclofenac, high gastrointestinal and cardiovascular risk)

PSAs were performed, the cost-effectiveness scatterplot of the comparison of two single drugs: imrecoxib vs. diclofenac was performed. It showed that there were more plots to the right of the cost-effectiveness threshold of $30,000 (3.0 GDP per capita approximately), diclofenac was dominated by imrecoxib with the probability of 99.71% and 99.35% in low and high GI and CV risk group, respectively (Figs. 1 and 2).

**Fig. 3:**
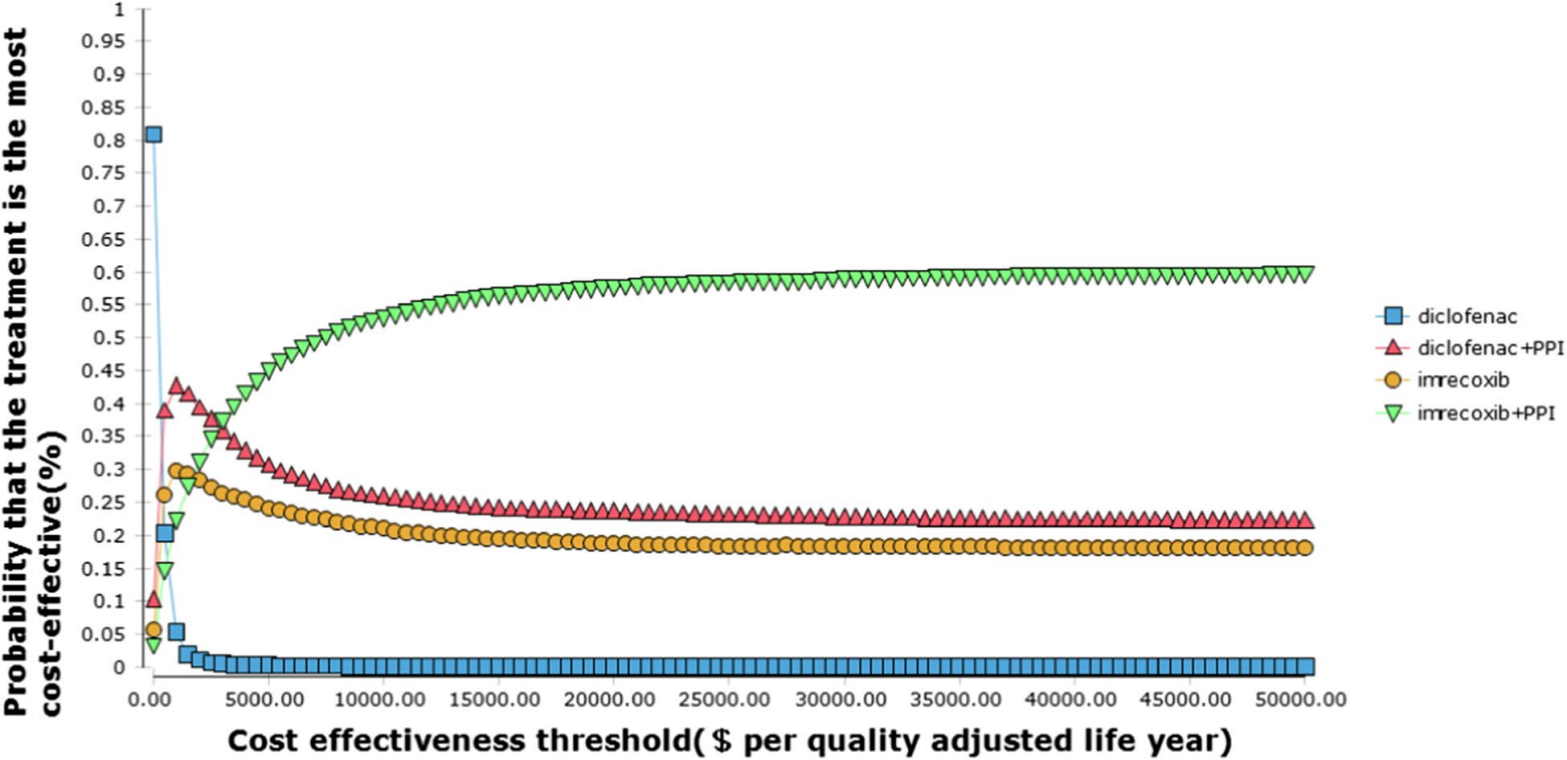
Cost and effectiveness acceptability curve (low gastrointestinal and cardiovascular risk)

**Fig. 4:**
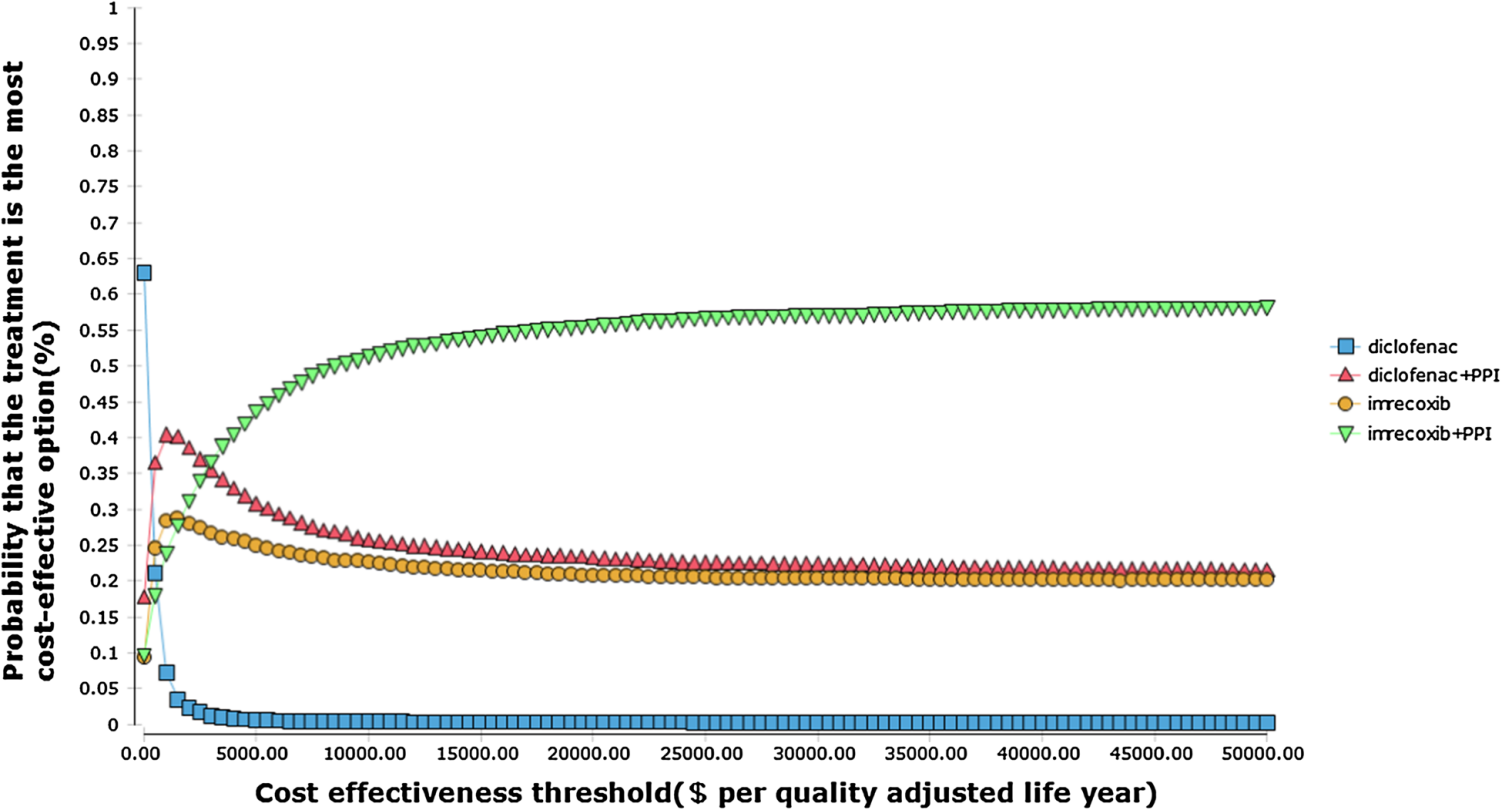
Cost and effectiveness acceptability curve (high gastrointestinal and cardiovascular risk)

The results of the PSA are shown, it suggested that in the low GI and CV risk group, for cost-effectiveness threshold below $500, diclofenac has the highest probability to be the most cost-effective option; for cost-effectiveness threshold between $500 and $3000, co-prescription of diclofenac and PPI was the most cost-effective option; for cost-effectiveness threshold above $3000, the co-prescription of imrecoxib and PPI has the highest probability to be the most cost-effective option. In high GI and CV risk group, for cost-effectiveness threshold below $300, diclofenac was the most cost-effective option, for cost-effectiveness threshold between $300 and $2500, co-prescription of diclofenac and PPI was likely to be the most cost-effective option; for cost-effectiveness threshold above $2500, co-prescription of imrecoxib and PPI has the highest probability to be the most cost-effective option (Figs. 3 and 4).

Using the threshold of $30,000 (3.0 GDP per capita approximately), there were 59.04% and 57.16% probability for imrecoxib plus PPI to be the most cost-effective option in the low and high GI and CV risk group, respectively.

## Discussion

To our knowledge, this is the first study to explore the cost effectiveness of imrecoxib and diclofenac, and the addition of a PPI to both treatment strategies. A Markov model based on the NICE OA model was used in the present study. DSA was performed to explore the robustness of model with one parameter changing according to its preset range. PSA was performed to explore the impact of joint uncertainties of model parameters: costs, utilities and transition probabilities. The results from the PSAs can provide more results compared with base-case analyses and DSAs [19, 23].

Of the four treatment strategies, none were strictly dominated by any other strategy in the base case analysis. Using the cost-effectiveness threshold $10,000 (1.0 GDP per capita approximately), the additions of a PPI to both imrecoxib and diclofenac were more cost-effective (especially for diclofenac) in the long-term use, which was similar to other reports on the cost-effectiveness of adding a PPI to NSAIDs or selective COX-2 inhibitors [5]. The cost-effectiveness of the addition of a PPI can also be found in patients at both low GI and CV risk, which provides a good suggestion for clinicians to prescript a PPI when prescribing imrecoxib and diclofenac, even for patients at low GI and CV risks. It can be a good way to save money to co-prescribe a PPI to imrecoxib and diclofenac based on the present findings.

When it comes to single drug, diclofenac was highly dominated by imrecoxib, it was more likely for imrecoxib to be the cost-effective option compared to diclofenac. The anti-inflammatory mechanism of NSAIDs is to inhibit the cyclooxygenase (COX), which is a requirement for prostaglandin synthesis [3]. There are mainly two types of COX: COX-1 and COX-2, COX-1 is involved in platelet activation, gastrointestinal protection and kidney function, and COX-2 is involved in inflammatory. Traditional NSAIDs can inhibit both COX-1 and COX-2, causing the GI toxicity, while selective COX-2 inhibitors can selectively inhibit COX-2 [6, 24]. From the perspective of mechanism, the lower GI events incidence rate of imrecoxib compared to diclofenac can be explained [6, 25]. Taking both price and incidence of AEs, the relatively high cost-effectiveness of imrecoxib can be obtained also with higher price compared to diclofenac. In China, according to the medical insurance policies, diclofenac was listed as first-line drug with higher reimbursement ratio compared to imrecoxib, which was listed as second-line drug [26]. From the perspective of long-term cost-effectiveness, higher reimbursement ratio of imrecoxib can be expected to encourage the wide use, because it’s a way to save money from the perspective of long-term cost-effectiveness, especially with the increase number of OA patients in China nowadays [1].

Although uncertainties existed in the present study, the results in the base-case analyses were robust to sensitivity analyses. According to the WHO-CHOICE recommendations, if the ICER < 1.0 GDP per capita (approximately $10,000 in China), the treatment was highly more cost-effective compared to another, if the ICER < 3.0 GDP per capita, the treatment was more cost-effective compared to another [27]. In the present study, with the changes of parameters according to their wide ranges in the DSAs, the ICERs all below $15,000 (1.5 GDP per capita approximately). In the PSAs, using the threshold of $30,000 (3.0 GDP per capita approximately), co-prescription of imrecoxib and a PPI had the highest probability to be the most cost-effective option, and diclofenac was high dominated by imrecoxib, which were robust to the results of PSAs.

There are several limitations of this study, first, as with all modeling studies, standard treatment was assumed for all patients when they suffered osteoarthritis or complied with other AEs. In real world, patients may change their original treatment option to another due to different reasons, for example, it was assumed that when patients moved to post AE states and post treatment states, they would stop the original medication and use topical diclofenac instead, but in real world there are many other options for patients. However, it’s also because of the preset standard treatment, the comparison of cost-effectiveness of different treatment strategies can be achieved. Second, there were large RCTs comparing the efficacy and safety of celecoxib and diclofenac, while there were limited RCTs comparing imrecoxib and celecoxib, imrecoxib and diclofenac. In order to decrease the effect of relative risk of probability of AEs of imrecoxib and celecoxib, DSA was performed to explore the influence of relative risk of probabilities of AEs of imrecoxib vs. celecoxib, the results stay robust with the wide range of relative risk of probabilities of AEs between imrecoxib and diclofenac. Third, when adapting the model to a Chinese perspective, a part of data was collected from the NICE model, which may not the same as that in the Chinese population. In order to decrease the uncertainties, Chinese real-world data was used to represent cost, general population mortality rate was collected from Chinese Yearbook to decrease the uncertainties caused by the source of parameters input.

## Conclusion

Although uncertainties in the model exist, based on our findings, it was suggested that a PPI can be added when prescribing imrecoxib or diclofenac (especially for diclofenac) to manage OA in the long-term use due to the high cost-effectiveness of a co-prescription of a PPI obtained in the present study, even for patients at low GI and CV risks. Imrecoxib provides a valuable treatment option, clinicians can consider using imrecoxib, and a higher reimbursement ratio of imrecoxib is expected to encourage the use of imrecoxib.

## Supplementary Information

**Additional file 1.** Additional tables/figures.

## Data Availability

The model, data of utility values, drug cost and probabilities were all extracted from existing literature. The data of clinical cost was extracted from the Shanghai Suvalue Health Scientific Ltd.

## Abbreviations

OA: Osteoarthritis
CV: Cardiovascular disease
NSAIDs: Non-steroidal anti-inflammatory drugs
COX: Cyclooxygenase
GI: Gastrointestinal
CDFA: Chinese Food and Drug Administration
NICE: National Institute for Health and Care Excellence
COX-2s: COX-2 inhibitors
PPI: Proton pump inhibitor
QALYs: Quality adjusted life years
ICER: Incremental cost-effectiveness ratio
OD: Once a day
TD: Three times a day
vs: Versus
MI: Myocardial infarct
HF: Heart failure
CPI: Consumer price index
AE: AE
HIS: Hospital Information System
RCT: Random control trial
DSA: Deterministic sensitivity analysis
PSA: Probability sensitivity analysis
AAOS: American Academy of Orthopedic Surgeons
OARSI: Osteoarthritis Research Society International

## Code availability

Code of Treeage, TN22N-881WS-14NHO-N.
