# Supplementary material for: Cost-utility analysis of imrecoxib compared with diclofenac for patients with osteoarthritis
Source: Cost Eff Resour Alloc. 2021 Apr 20;19:22. doi: 10.1186/s12962-021-00275-7 (PMC8056517; doi:10.1186/s12962-021-00275-7)

**Additional file**

**Figure A1. Overview of the OA model**


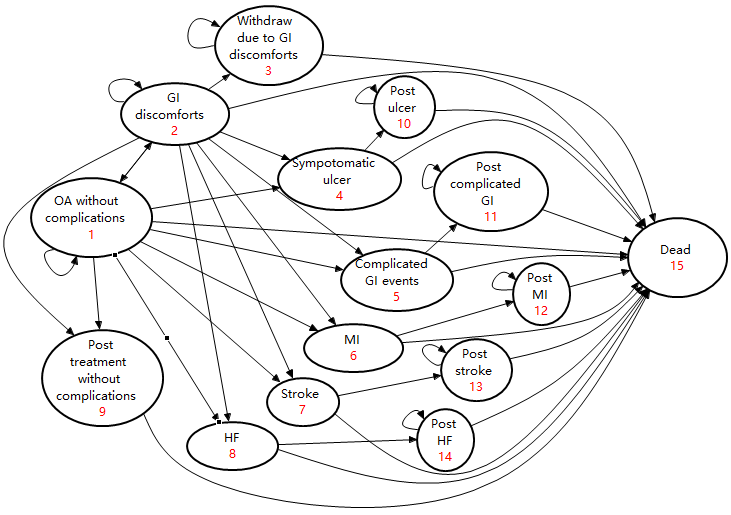


**Table A1. Cost of omeprazole**

| **No.** | **Current names** | **Trade name** | **Specifications** | **Manufacturer** | **Price per box (RMB)** | **Recommended dose** | **Cost per day (RMB)** |
| --- | --- | --- | --- | --- | --- | --- | --- |
| 1 | Omprazole enteric-coated tablets | ---- | 10mg, 36 capsules per box | Shandong new era Pharmaceutical Co., Ltd | 48.98 | 20-40mg per day | 2.72-5.44 |
| 2 | Omeprazole enteric-coated tablets | Kunliyu | 20mg per capsules, 28 capsules per box | Beijing Taiyang Pharmaceutical Co., Ltd | 9.91 | 20-40mg per day | 0.36-0.71 |
| 3 | Omeprazole enteric-coated tablets | Jin’aokang | 20mg per capsules, 14 capsules per box | Zhejiang Jinhua Kangenbei biopharmaceutical Co., Ltd | 56.06 | 20-40mg | 7.14-14.29 |
| 4 | Esomeprazole magnesium enteric-coated tablets | Naixin | 20mg per capsules, 7 capsules per box | AstraZeneca Pharmaceutical Co., Ltd | 61.76 | 20-40mg | 8.82-17.65 |
| 5 | Esomeprazole magnesium enteric-coated tablets | Naixin | 40 mg per capsules, 7 capsules per box | AstraZeneca Pharmaceutical Co., Ltd | 105 | 20-40mg | 7.5-15 |
| 6 | Omeprazole magnesium enteric-coated tablets | ---- | 10 mg per capsules, 14 capsules per box | Xuchang high tech Pharmaceutical Co., Ltd | 51.72 | 20mg | 7.39 |
| 7 | Omeprazole magnesium enteric-coated tablets | ---- | 10 mg per capsules, 7 capsules per box | Xuchang high tech Pharmaceutical Co., Ltd | 26.09 | 20mg | 7.45 |
| 8 | Omeprazole enteric-coated tablets | Shuangjingjili | 20 mg per capsules, 28 capsules per box | Qingdao Shuangjing Pharmaceutical Co., Ltd | 25.48 | 20-40mg | 0.91-1.82 |
| 9 | Omeprazole enteric capsules | ---- | 20mg per capsule, 21 capsules per box | Yangzi River Pharmaceutical Group Co., Ltd | 30.38 | 20-40mg | 1.45-2.89 |
| 10 | Omeprazole enteric capsules | ---- | 20mg per capsule, 28 capsules per box | CommScope Pharmaceutical Co., Ltd | 17.50 | 20-40mg | 0.63-1.25 |
| 11 | Omeprazole enteric-coated tablets | ---- | 10mg per capsule, 36 capsules per box | Xinhe Yuansheng Pharmaceutical Co., Ltd | 12.07 | 40-80mg | 1.34-2.68 |
| 12 | Omeprazole sodium enteric-coated tablets | Liweiting | 10mg per capsule, 28 capsules per box | Suzhou Sinochem Pharmaceutical Industry Co., Ltd | 6.7 | 20-40mg | 0.48-0.96 |
| 13 | Omeprazole enteric capsules | ---- | 20mg per capsule, 28 capsules per box | Hainan huluwa Pharmaceutical Group Co., Ltd | 49 | 20-40mg | 1.75-3.50 |
| 14 | Omeprazole and sodium bicarbonate capsules | ---- | 20mg per capsule, 7 capsules per box | Xiamen Ensheng Pharmaceutical Co., Ltd | 85.60 | 20mg | 12.23 |
| 15 | Esomeprazole enteric casules | Laimeishu | 20mg per capsule, 7 capsules per box | Chongqing Laimei Pharmaceutical Co., Ltd | 64.98 | 20mg | 9.28 |
| 16 | Omeprazole enteric capsules | ---- | 20mg per capsule, 14 capsules per box | Wanbond Pharmaceutical Group Co., Ltd | 19.60 | 20-40mg | 1.40-2.80 |
| 17 | Omeprazole enteric capsules | ---- | 20mg per capsule, 14 capsules per box | CommScope Pharmaceutical Co., Ltd | 10.8 | 20-40mg | 0.77-1.54 |
| 18 | Omeprazole enteric0coated capsules | Aoke | 20mg per capsule, 7 capsules per box | Changzhou siyao Pharmaceutical Co., Ltd | 19 | 20-40mg | 2.71-5.43 |
| 19 | Omeprazole enteric0coated capsules | Aoke | 20mg per capsule, 14 capsules per box | Changzhou siyao Pharmaceutical Co., Ltd | 38 | 20-40mg | 2.71-5.43 |
| 20 | Omeprazole magnesium enteric-coated tablets | Luosaike MUPS | 10mg per capsule, 7 capsules per box | AstraZeneca Pharmaceutical Co., Ltd | 41.62 | 20-40mg | 11.89-23.78 |
| 21 | Omeprazole magnesium enteric-coated tablets | Luosaike MUPS | 20mg per capsule, 7 capsules per box | AstraZeneca Pharmaceutical Co., Ltd | 72.93 | 20-40mg | 10.42-20.84 |
| 22 | Omeprazole magnesium enteric-coated tablets | Luosaike MUPS | 20mg per capsule, 14 capsules per box | AstraZeneca Pharmaceutical Co., Ltd | 145.05 | 20-40mg | 10.36-20.72 |
| 23 | Omeprazole enteric-coated tablets | Shuangjingjili | 10mg per capsule, 28 capsules per box | Qingdao Shuangjing Pharmaceutical Co., Ltd | 10.07 | 20-40mg | 0.72-1.44 |
| 24 | Omeprazole magnesium enteric-coated tablets | ---- | 20mg per capsule, 7 capsules per box | Xuchang high tech Pharmaceutical Co., Ltd | 49.98 | 20mg | 7.14 |
| Average cost per day (20mg) | | | | | | | 4.87 |

Overall, the cost of omeprazole is 4.87 RMB per day, because the RMB exchange rate against the USD was 100:689.85 in 2019, the cost of omeprazole is ＄0.71 per day, it was supposed that the treatment duration of using omeprazole was 4 weeks during each circle, in each circle, the cost of omeprazole was set to ＄19.88.

**Table A2. Cost of imrecoxib and diclofenac**

| **No.** | **Current names** | **Trade name** | **Specifications** | **Manufacturer** | **Price per box (RMB)** | **Recommended dose** | **Cost per day (RMB)** |
| --- | --- | --- | --- | --- | --- | --- | --- |
| 1 | Imrecoxib | Hengyang | 100mg per capsule, 10 capsules per box | Jiangsu Hengrui Pharmaceutical Co., Ltd | 49 | 0.2g per day, 8 weeks | 9.8 |
| 2 | Diclofenac sodium dual release enteric-coated capsules | Daifen | 75mg per capsule, 20 capsule per box | Temmler Ireland Ltd. | 38.64 | 75mg-150mg | 3.86 (150mg) |
| 3 | Diclofenac sodium enteric-coated tablets | ---- | 25mg per capsule, 24 capsules per box | Baiyunshan Tangyin Dongtai Pharmaceutical Co., Ltd | 1.5 | 75mg-150mg | 0.38 (150mg) |
| 4 | Diclofenac sodium enteric-coated sustained release capsules | Nuofuding | 100mg per capsule, 16 capsules per box | Hainan Puli Pharmaceutical Co., Ltd | 22.80 | 100mg | 2.14 (150mg) |
| 5 | Diclofenac sodium sustained-release capsules | ---- | 50mg per capsule, 36 capsules per box | Nanjing Yiheng Pharmaceutical Co., Ltd | 27.00 | 100mg | 2.25 (150mg) |
| 6 | Diclofenac sodium sustained release capsules | Yingtaiqing | 50mg per capsule, 20 capsules per box | Yaoda Pharmaceutical Co., Ltd | 14.67 | 100mg | 2.20 (150mg) |
| 7 | Diclofenac sodium enteric-coated tablets | Futaling | 25mg/30 | Beijing novartis pharma ltd | 16.85 | 100-150mg | 3.37 (150mg) |
| Average cost of imrecoxib per day (200mg daily) | | | | | | | 9.80 |
| Average cost of diclofenac per day (150mg daily) | | | | | | | 2.37 |

Overall, the cost of imrecoxib and diclofenac were 9.80 RMB (＄1.42) and 2.37 RMB (＄0.34), respectively. RMB exchange rate against the USD was 100:689.85. It was assumed that in each circle, the cost were ＄19.89 and ＄4.81 for imrecoxib and diclofenac, respectively.

**Table A3. Cost of topical diclofenac**

| **Current names** | **Trade name** | **Specifications** | **Manufacturer** | **Price per box (RMB)** | **Recommended dose** | **Cost per day (RMB)** |
| --- | --- | --- | --- | --- | --- | --- |
| Topical diclofenac | Lai Bixin | 4 pieces per box | Bengbu Fengyuan Tushan Pharmaceutical Co., Ltd | 45.00 | 1 piece per day | 11.25 |
| Topical diclofenac | Lai Bixin | 6 pieces per box | Bengbu Fengyuan Tushan Pharmaceutical Co., Ltd | 55.00 | 1 piece per day | 9.17 |
| Average cost of topical diclofenac per day (1 piece per day) | | | | | | 10.00 |

Overall, the cost of topical diclofenac per day was 10.00 RMB (＄1.45), RMB exchange rate against the USD was 100:689.85. It was assumed that in each circle, the cost of topical diclofenac was ＄20.30.

**Table A4. Cost of adverse events**

| **Adverse events** | **Per outpatient visiting (＄)** | **Per inpatient visiting (＄)** | **Assumption** | **Cost input in each circle (＄)** |
| --- | --- | --- | --- | --- |
| GI discomforts | 10.52 | ——— | In each circle, each patient in “GI discomforts” state will have an outpatient visiting, and will not stop the medication of OA | 10.52+cost of managing OA |
| Symptomatic ulcer | 34.57 | ——— | In each circle, each patient in “Symptomatic ulcer” state will have an outpatient visiting | 34.57 |
| Complicated GI | 43.41 | 1310.74 | In each circle, each patient in “Complicated GI” state will have both an outpatient visiting and inpatient visiting | 1354.15 |
| Stroke | 49.22 | 1240.70 | In each circle, each patient in “Stroke” state was assumed to have one outpatient visiting and inpatient visiting | 1289.92 |
| HF | 32.81 | 1149.52 | In each circle, each patient in “HF” state was assumed to have one outpatient visiting and inpatient visiting | 1182.33 |
| MI | 111.36 | 5079.56 | In each circle, each patient in “MI” state was assumed to have one outpatient visiting and inpatient visiting | 5190.93 |

**RMB exchange rate against the USD was 100:689.85 and the consumer price index (CPI) was 101.6%, 102.1% and 102.9% in 2017, 2018, and 2019, respectively. All the cost was adjusted to 2019 based on exchange rate and CPI.*

**Table A5. Cost of managing OA, including the items of consulting fee, examination fee, treatment fee, materials fee, and other fee per visiting**

| Number of patients | 1580 | | |
| --- | --- | --- | --- |
| Number of outpatients visiting | 2884 | | |
| Fees | Mean (＄) | Proportion (%) | Cost in each circle (without Chinese and Western medicine) |
| Consulting fee | 1.55 | 3.39 | 26.50 |
| Treatment fee | 8.06 | 17.58 |  |
| Chinese medicine fee | 6.40 | 13.96 |  |
| Western medicine fee | 12.95 | 28.24 |  |
| Materials fee | 1.06 | 2.30 |  |
| Examination fee | 14.82 | 32.32 |  |
| Laboratory fee | 0.99 | 2.16 |  |
| Others | 0.02 | 0.04 |  |
| Total | 45.85 | 100 |  |

**RMB exchange rate against the USD was 100:689.85 and the consumer price index (CPI) was 101.6%, 102.1% and 102.9% in 2017, 2018, and 2019, respectively. All the cost was adjusted to 2019 based on exchange rate and CPI.*

There’s an assumption that in each circle, OA patients would have **one outpatient visiting** on average. Cost of managing OA in each circle is calculated by using the average cost per outpatient visiting (consulting fee, treatment fee, materials fee, examination fee, laboratory fee and other fee, except for the drug fee) and the price of specific drug.

**Table A6. Mortality probabilities of formal patients and patients with adverse events**

| **Groups** | **Probabilities (%)** | **Source** |
| --- | --- | --- |
| **General population** | | |
| 55-64 | 0.37 | Chinese Health Yearbook [1] |
| 65-74 | 0.94 | Chinese Health Yearbook [1] |
| 75-84 | 2.67 | Chinese Health Yearbook [1] |
| 85+ | 5.14 | Chinese Health Yearbook [1] |
| **Patients with adverse events** | | |
| Complicated GI events | 9.51 | NICE Guideline [2] |
| Post Complicated GI events | 2.77 | NICE Guideline [2] |
| MI | 4.28 | NICE Guideline [2] |
| Post MI | 0.45 | NICE Guideline [2] |
| Stroke | 3.23 | NICE Guideline [2] |
| Post stroke | 0.46 | NICE Guideline [2] |
| HF | 4.95 | NICE Guideline [2] |

There’s assumption that the patients with GI discomforts, ulcer and post ulcer have the same mortality rate with general population.

**Table A7. CHEERS Checklist**

| Section/item | No. | Recommendation | Reported in study |
| --- | --- | --- | --- |
| **Title and abstract** | | | |
| Title | 1 | Identify the study as an economic evaluation or use more specific terms such as “cost-effectiveness analysis”, and describe the interventions compared. | Page 1, line 1-2 |
| Abstract | 2 | Provide a structured summary of objectives, perspective, setting, methods (including study design and inputs), results (including base case and uncertainty analyses), and conclusions. | Page 2, line 1-21 |
| **Introduction** |  |  |  |
| Background and objectives | 3 | Provide an explicit statement of the broader context for the study. Present the study question and its relevance for health policy or practice decisions. | Page3-4 |
| **Methods** | | | |
| Target population and subgroups | 4 | Describe characteristics of the base case population and subgroups analysed, including why they were chosen. | Page 7, line 9-14 |
| Setting and location | 5 | State relevant aspects of the system(s) in which the decision(s) need(s) to be made. | Page 4, line 18-20 |
| Study perspective | 6 | Describe the perspective of the study and relate this to the costs being evaluated. | Page 4, line 20 |
| Comparators | 7 | Describe the interventions or strategies being compared and state why they were chosen. | Page 5, line 10-Page 6, line 4 |
| Time horizon | 8 | State the time horizon(s) over which costs and consequences are being evaluated and say why appropriate. | Page 6 line 20- Page 7 line 6 |
| Discount rate | 9 | Report the choice of discount rate(s) used for costs and outcomes and say why appropriate. | Page 7, line 2 |
| Choice of health outcomes | 10 | Describe what outcomes were used as the measure(s) of benefit in the evaluation and their relevance for the type of analysis performed. | Page 8, line 21- Page 9, line 6 |
| Measurement of effectiveness | 11a | Single study-based estimates: Describe fully the design features of the single effectiveness study and why the single study was a sufficient source of clinical effectiveness data. | Page 8, line 21- Page 9, line 6 |
|  | 11b | Synthesis-based estimates: Describe fully the methods used for identification of included studies and synthesis of clinical effectiveness data. | Not applicable |
| Measurement and valuation of preference-based outcomes | 12 | If applicable, describe the population and methods used to elicit preferences for outcomes. | Not applicable |
| Estimating resources and costs | 13a | Single study-based economic evaluation: Describe approaches used to estimate resource use associated with the alternative interventions. Describe primary or secondary research methods for valuing each resource item in terms of its unit cost. Describe any adjustments made to approximate to opportunity cost. | Page 7, line 15-Page 8, line 19 |
|  | 13b | Model-based economic evaluation: Describe approaches and data sources used to estimate resource use associated with model health states. Describe primary or secondary research methods for valuing each resource item in terms of its unit cost. Describe any adjustments made to approximate to opportunity costs. | Not applicable |
| Currency, price date, and conversion | 14 | Report the dates of the estimated resource quantities and unit costs. Describe methods for adjusting estimated unit costs to the year of reported costs if necessary. Describe methods for converting costs into a common currency base and the exchange rate. | Page 7, line 16-20; Additional file: Figure A1-A5 |
| Choice of model | 15 | Describe and give reasons for the specific type of decision analytical model used. Providing a figure to show model structure is strongly recommended. | Page 3, line 19-Page 4, line 6; Additional file: Figure A1 |
| Assumptions | 16 | Describe all structural or other assumptions underpinning the decision-analytical model. | Page 6, line 8-19 |
| Analytical methods | 17 | Describe all analytical methods supporting the evaluation. This could include methods for dealing with skewed, missing, or censored data; extrapolation methods; methods for pooling data; approaches to validate or make adjustments (such as half cycle corrections) to a model; and methods for handling population heterogeneity and uncertainty. | Page 6, line 20- Page 7, line 8 |
| **Results** | | | |
| Study parameters | 18 | Report the values, ranges, references, and, if used, probability distributions for all parameters. Report reasons or sources for distributions used to represent uncertainty where appropriate. Providing a table to show the input values is strongly recommended. | Table 1, Additional file: Table A8 |
| Incremental costs and outcomes | 19 | For each intervention, report mean values for the main categories of estimated costs and outcomes of interest, as well as mean differences between the comparator groups. If applicable, report incremental cost-effectiveness ratios. | Page 11, line 7-19, Table 2 |
| Characterizing uncertainty | 20a | Single study-based economic evaluation: Describe the effects of sampling uncertainty for the estimated incremental cost and incremental effectiveness parameters, together with the impact of methodological assumptions (such as discount rate, study perspective). | Not applicable |
|  | 20b | Model-based economic evaluation: Describe the effects on the results of uncertainty for all input parameters, and uncertainty related to the structure of the model and assumptions. | Page 11, line 20-Page 13, line 13 |
| Characterizing heterogeneity | 21 | If applicable, report differences in costs, outcomes, or cost-effectiveness that can be explained by variations between subgroups of patients with different baseline characteristics or other observed variability in effects that are not reducible by more information. | Not applicable |
| **Discussion** |  |  |  |
| Study findings, limitations, generalizability, and current knowledge | 22 | Summarize key study findings and describe how they support the conclusions reached. Discuss limitations and the generalizability of the findings and how the findings fit with current knowledge. | Page 13: line15-22, Page15: line 20-Page 16: line 18 |
| **Other** | | | |
| Source of funding | 23 | Describe how the study was funded and the role of the funder in the identification, design, conduct, and reporting of the analysis. Describe other non-monetary sources of support. | Page 18, line 6 |
| Conflicts of interest | 24 | Describe any potential for conflict of interest of study contributors in accordance with journal policy. In the absence of a journal policy, we recommend authors comply with International Committee of Medical Journal Editors recommendations. | Page 18, line 4-5 |

**Table A8. Distributional information for model inputs**

| **Variables Name** | **Distribution** | **Estimate** | **α** | **β** | **Median** | **SE** | **95%CI** | |
| --- | --- | --- | --- | --- | --- | --- | --- | --- |
|  |  |  |  |  |  |  | **Lower** | **Upper** |
| Cost-diclofenac | Gamma | 4.8100 |  |  |  | 1.2270 | 2.4050 | 7.2150 |
| Cost-imrecoxib | Gamma | 19.8900 |  |  |  | 5.0740 | 9.9450 | 29.8350 |
| Cost-omeprazole | Gamma | 19.8800 |  |  |  | 5.0714 | 9.9400 | 29.8200 |
| Cost-topical diclofenac | Gamma | 20.3000 |  |  |  | 5.1786 | 10.1500 | 30.4500 |
| Cost-Managing OA without any drugs | Gamma | 26.5000 |  |  |  | 6.7602 | 13.2500 | 39.7500 |
| Cost-GI | Gamma | 10.5200 |  |  |  | 2.6837 | 5.2600 | 15.7800 |
| Cost-Ulcer | Gamma | 34.5700 |  |  |  | 8.8189 | 17.2850 | 51.8550 |
| Cost-Complicated GI | Gamma | 1354.1500 |  |  |  | 345.4464 | 677.0750 | 2031.2250 |
| Cost-Stroke | Gamma | 1289.9200 |  |  |  | 329.0612 | 644.9600 | 1934.8800 |
| Cost-MI | Gamma | 5190.9300 |  |  |  | 1324.2168 | 2595.4650 | 7786.3950 |
| Cost-HF | Gamma | 1182.3300 |  |  |  | 301.6148 | 591.1650 | 1773.4950 |
| Cost-Post ulcer | Gamma | 0.8100 |  |  |  | 0.2066 | 0.4050 | 1.2150 |
| Cost-Post complicated GI | Gamma | 21.5400 |  |  |  | 5.4949 | 10.7700 | 32.3100 |
| Cost-Post stroke | Gamma | 619.5900 |  |  |  | 158.0587 | 309.7950 | 929.3850 |
| Cost-Post MI | Gamma | 948.4700 |  |  |  | 241.9566 | 474.2350 | 1422.7050 |
| Cost-Post HF | Normal | 451.2900 |  |  |  | 115.1250 | 225.6450 | 676.9350 |
| Probability-Diclofenac-GI | Beta | 0.2130 | 903.5460 | 3338.4540 |  |  | 0.1065 | 0.3195 |
| Probability-Diclofenac-Ulcer | Beta | 0.0014 | 5.9388 | 4236.0612 |  |  | 0.0007 | 0.0021 |
| Probability-Diclofenac-Complicated GI | Beta | 0.0007 | 2.9694 | 4239.0306 |  |  | 0.0004 | 0.0011 |
| Probability-Diclofenac-Stroke | Beta | 0.0006 | 2.5452 | 4239.4548 |  |  | 0.0003 | 0.0009 |
| Probability-Diclofenac-MI | Beta | 0.0009 | 3.8178 | 4238.1822 |  |  | 0.0005 | 0.0014 |
| Probability-Diclofenac-HF | Beta | 0.0002 | 0.8484 | 4241.1516 |  |  | 0.0001 | 0.0003 |
| Probability-general-death [Age years=55-64] | Uniform | 0.0037 |  |  |  |  | 0.0019 | 0.0056 |
| Probability-general-death [Age years=65-74] | Uniform | 0.0094 |  |  |  |  | 0.0047 | 0.0141 |
| Probability-general-death [Age years=75-80] | Uniform | 0.0267 |  |  |  |  | 0.0134 | 0.0401 |
| Probability-Complicated GI-death | Uniform | 0.0951 |  |  |  |  | 0.0476 | 0.1427 |
| Probability-Post Complicated GI-death | Uniform | 0.0277 |  |  |  |  | 0.0139 | 0.0416 |
| Probability-stroke-death | Uniform | 0.0323 |  |  |  |  | 0.0162 | 0.0485 |
| Probability-Post stroke-death | Uniform | 0.0046 |  |  |  |  | 0.0023 | 0.0069 |
| Probability-HF-death | Uniform | 0.0495 |  |  |  |  | 0.0248 | 0.0743 |
| Probability-Post HF-death | Uniform | 0.0017 |  |  |  |  | 0.0009 | 0.0026 |
| Probability-MI-death | Uniform | 0.0428 |  |  |  |  | 0.0214 | 0.0642 |
| Probability-Post MI-death | Uniform | 0.0045 |  |  |  |  | 0.0023 | 0.0068 |
| Probability-COX-withdraw | Beta | 0.1120 | 446.5440 | 3540.4560 |  |  | 0.0560 | 0.1680 |
| Probability-NSAID-withdraw | Beta | 0.1390 | 553.3590 | 3427.6140 |  |  | 0.0695 | 0.2085 |
| Utility-OA patients with treatment of NSAID/COX | Beta | 0.1806 |  |  |  | 0.0461 | 0.0903 | 0.2709 |
| Relative Risk-GI-Imrecoxib vs. Diclofenac | Lognormal | 0.3300 |  |  | 0.2640 |  | 0.1650 | 0.4950 |
| Relative Risk-Ulcer-Imrecoxib vs. Diclofenac | Lognormal | 0.5800 |  |  | 0.4640 |  | 0.2900 | 0.8700 |
| Relative Risk-Complicated GI-Imrecoxib vs. Diclofenac | Lognormal | 0.3400 |  |  | 0.2720 |  | 0.1700 | 0.5100 |
| Relative Risk-Stroke-Imrecoxib vs. Diclofenac | Lognormal | 0.5100 |  |  | 0.4080 |  | 0.2550 | 0.7650 |
| Relative Risk-MI-Imrecoxib vs. Diclofenac | Lognormal | 1.4000 |  |  | 1.1200 |  | 0.7000 | 2.1000 |
| Relative Risk-HF-Imrecoxib vs. Diclofenac | Lognormal | 1.4200 |  |  | 1.1360 |  | 0.7100 | 2.1300 |
| Relative Risk-Probability to GI-COX+PPI vs. COX | Lognormal | 0.2400 |  |  | 0.1920 |  | 0.0300 | 0.7800 |
| Relative Risk-Probability to Ulcer-COX+PPI vs. COX | Lognormal | 0.2400 |  |  | 0.1920 |  | 0.0300 | 0.7800 |
| Relative Risk-Probability to Complicated GI-COX+PPI vs. COX | Lognormal | 0.2400 |  |  | 0.1920 |  | 0.0300 | 0.7800 |
| Relative Risk-Probability to Stroke-COX+PPI vs. COX | Lognormal | 1.0000 |  |  | 0.8000 |  | 0.5000 | 1.5000 |
| Relative Risk-Probability to MI-COX+PPI vs. COX | Lognormal | 1.0000 |  |  | 0.8000 |  | 0.5000 | 1.5000 |
| Relative Risk-Probability to HF-COX+PPI vs. COX | Lognormal | 1.0000 |  |  | 0.8000 |  | 0.5000 | 1.5000 |
| Relative Risk-Probability to GI-NSAID+PPI vs. NSAID | Lognormal | 0.4300 |  |  | 0.3440 |  | 0.2400 | 0.7600 |
| Relative Risk-Probability to Ulcer-NSAID+PPI vs. NSAID | Lognormal | 0.3700 |  |  | 0.2960 |  | 0.3000 | 0.4600 |
| Relative Risk-Probability to Complicated GI-NSAID+PPI vs. NSAID | Lognormal | 0.3200 |  |  | 0.2560 |  | 0.0700 | 2.9200 |
| Relative Risk-Probability to Stroke-NSAID+PPI vs. NSAID | Lognormal | 1.0000 |  |  | 0.8000 |  | 0.5000 | 1.5000 |
| Relative Risk-Probability to MI-NSAID+PPI vs. NSAID | Lognormal | 1.0000 |  |  | 0.8000 |  | 0.5000 | 1.5000 |
| Relative Risk-Probability to HF-NSAID+PPI vs. NSAID | Lognormal | 1.0000 |  |  | 0.8000 |  | 0.5000 | 1.5000 |
| Relative Risk-Utility-GI | Lognormal | 0.7330 |  |  | 0.5864 |  | 0.3665 | 1.0995 |
| Relative Risk-Utility-withdraw due to GI | Lognormal | 0.9890 |  |  | 0.7912 |  | 0.4945 | 1.4835 |
| Relative Risk-Utility-Ulcer | Lognormal | 0.5520 |  |  | 0.4416 |  | 0.2760 | 0.8280 |
| Relative Risk-Utility-Post Ulcer | Lognormal | 0.9800 |  |  | 0.7840 |  | 0.4900 | 1.4700 |
| Relative Risk-Utility-Complicated GI | Lognormal | 0.4590 |  |  | 0.3672 |  | 0.2295 | 0.6885 |
| Relative Risk-Utility-Post Complicated GI | Lognormal | 0.9800 |  |  | 0.7840 |  | 0.4900 | 1.4700 |
| Relative Risk-Utility-Stroke | Lognormal | 0.3480 |  |  | 0.2784 |  | 0.1740 | 0.5220 |
| Relative Risk-Utility-Post Stroke | Lognormal | 0.7060 |  |  | 0.5648 |  | 0.3530 | 1.0590 |
| Relative Risk-Utility-MI | Lognormal | 0.3740 |  |  | 0.2992 |  | 0.1870 | 0.5610 |
| Relative Risk-Utility-Post MI | Lognormal | 0.8800 |  |  | 0.7040 |  | 0.4400 | 1.3200 |
| Relative Risk-Utility-HF | Lognormal | 0.7100 |  |  | 0.5680 |  | 0.3550 | 1.0650 |
| Relative Risk-Utility-Post HF | Lognormal | 1.0000 |  |  | 0.8000 |  | 0.5000 | 1.5000 |
| Relative Risk-Utility-Post treatment | Lognormal | 0.9890 |  |  | 0.7912 |  | 0.4945 | 1.4835 |
| Relative Risk-risk of Bleeding/Ulcer [aged 65] vs. [aged 55] | Lognormal | 2.9600 |  |  | 2.3680 |  | 1.4800 | 4.4400 |
| Relative Risk-risk of CV [aged 65] vs. [aged 55] | Lognormal | 1.9400 |  |  | 1.5520 |  | 0.9700 | 2.9100 |

α and β are parameters of Beat distribution.

**Figure A2. Tornado diagram-Diclofenac + PPI vs. Diclofenac (patients aged 55 years old)**
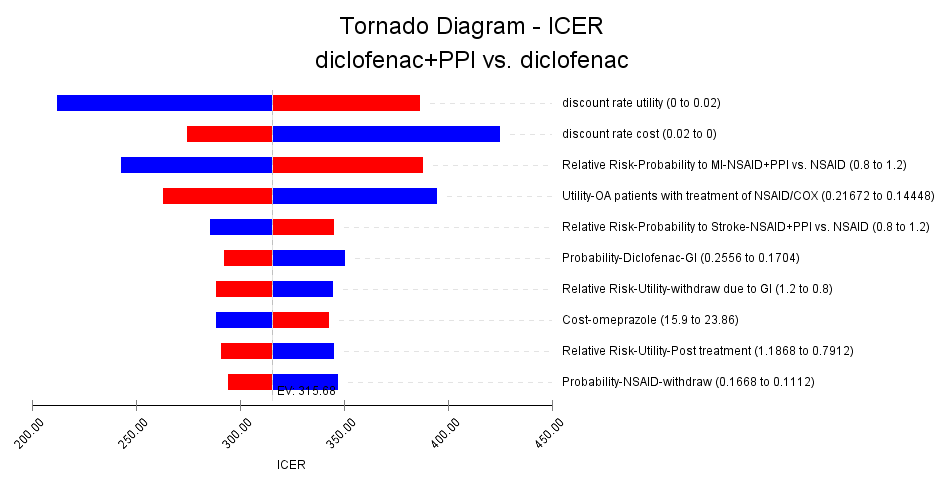


**Figure A3. Tornado diagram-Imrecoxib + PPI VS. Imrecoxib (patients aged 55 years old)**


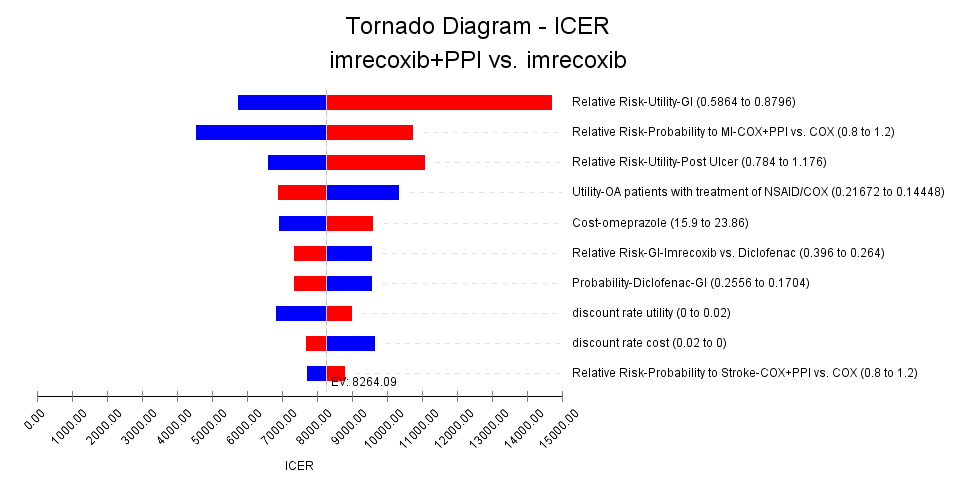


**Figure A4. Tornado diagram-Imrecoxib vs. Diclofenac (patients aged 55 years old)**


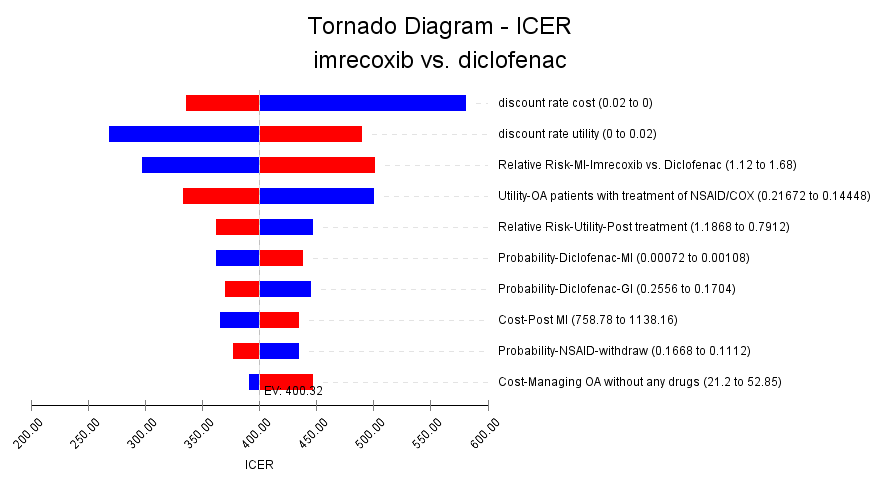


**Figure A5. Tornado diagram-Imrecoxib+PPI vs. Diclofenac+PPI (patients aged 55 years old)**


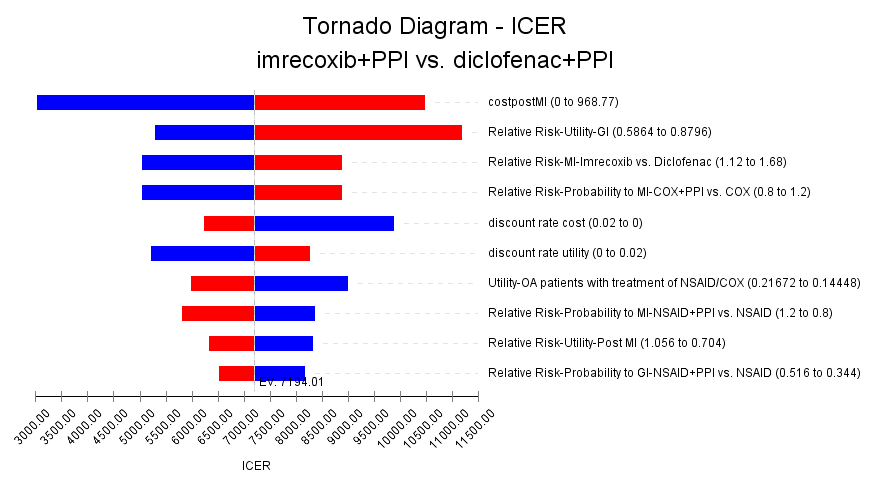


**Figure A6. Tornado diagram-Diclofenac + PPI vs. Diclofenac (patients aged 65 years old)**


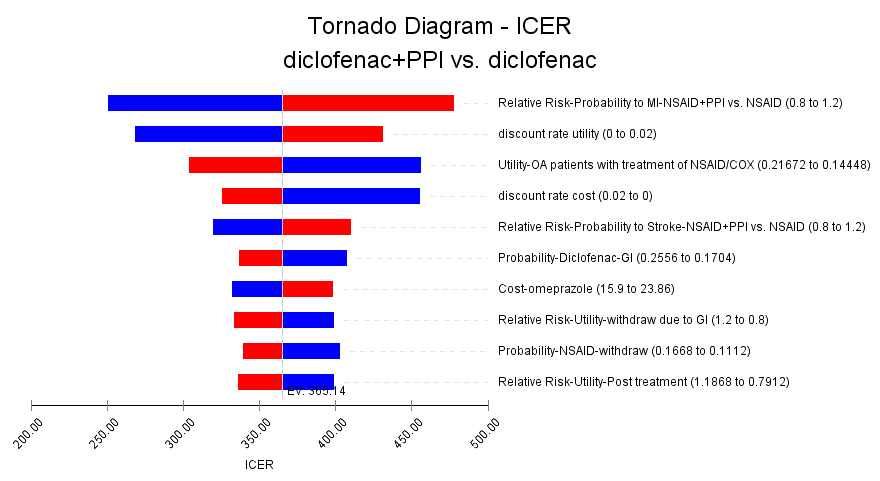


**Figure A7. Tornado diagram-Imrecoxib + PPI vs. Imrecoxib (patients aged 65 years old)**


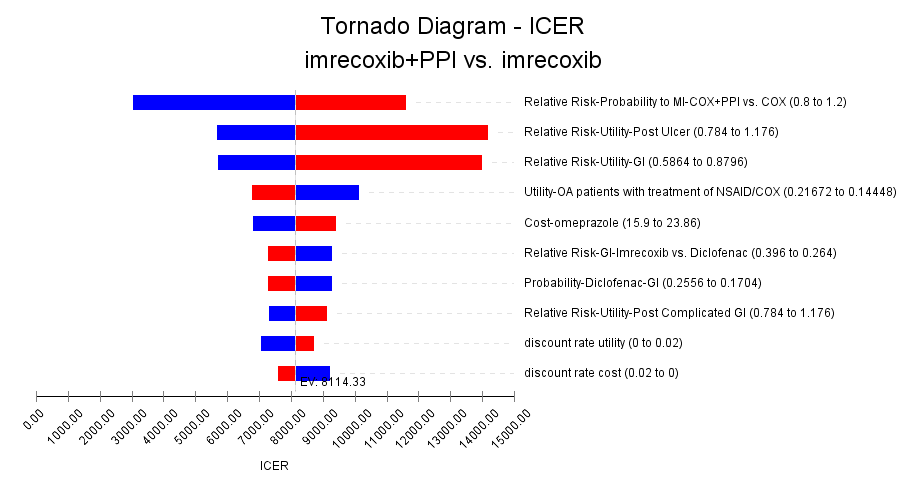


**Figure A8. Tornado diagram-Imrecoxib vs. Diclofenac (patients aged 65 years old)**


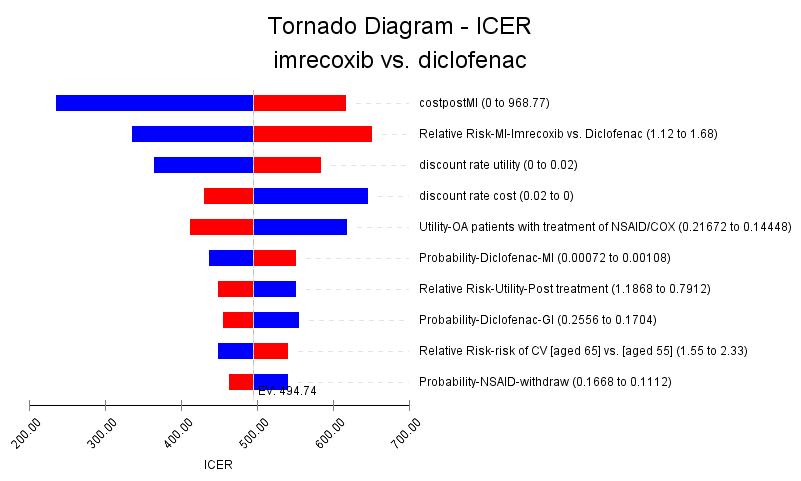


**Figure A9. Tornado diagram-Imrecoxib+PPI vs. Diclofenac+PPI (patients aged 65 years old)**


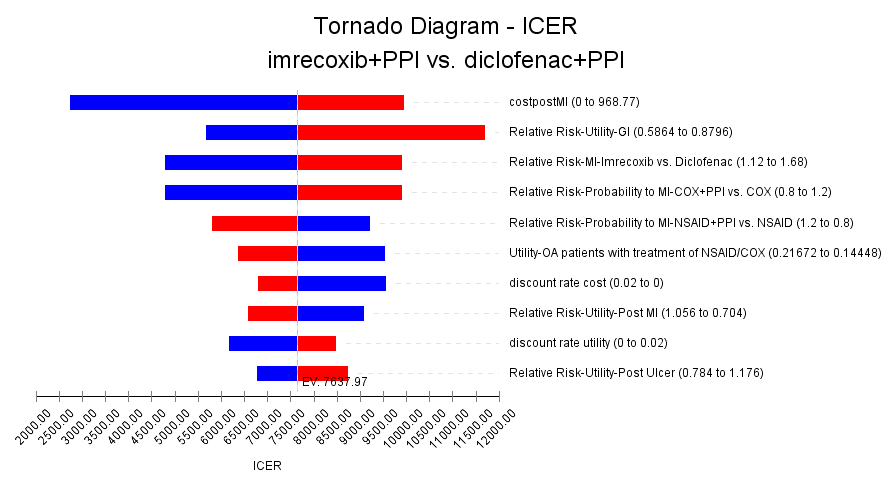

Supplement: Supplementary file 1 — Additional file 1. Additional tables/figures. [file 12962_2021_275_MOESM1_ESM.docx]
